# A Case of Immunotactoid Glomerulopathy with Rapid Progression to End-Stage Renal Disease

**DOI:** 10.1100/tsw.2009.164

**Published:** 2009-12-16

**Authors:** Shikha Jain, Darshika Chhabra

**Affiliations:** ^1^ Department of Internal Medicine, University of Illinois, Chicago, USA; ^2^ Department of Nephrology, Northwestern University, Chicago, USA

**Keywords:** immunotactoid glomerulopathy, acute kidney injury, end-stage renal disease, immunoglobulin

## Abstract

Immunotactoid glomerulopathy (IGN) is a rare immunoglobulin deposition disease. It is often mistaken for cryoglobulinemia or amyloidosis due to the similarities on biopsy findings. The disease progresses to end-stage renal disease (ESRD) within 7 months to 10 years. This is the first case reported of a patient with a diagnosis of IGN who developed acute kidney injury (AKI) and ESRD within 1 week of initial presentation.

## INTRODUCTION

Immunoglobulin (Ig)-mediated kidney disorders are divided into two categories: those that result from the deposition of intact Ig molecules into the kidney and those caused by deposition of components of Ig molecules, such as light or heavy chains or chain fragments. Disorders due to intact Ig deposition include IgA nephropathy, membranous lupus, and anti-GBM (glomerular basement membrane) disease. These pathologies are caused by polyclonal Ig molecules that interact directly with kidney antigens or form preformed immune complexes. In contrast, disorders due to deposition of Ig components are monoclonal, and the pathogenic proteins are produced by clonal populations of plasma cells or B lymphocytes. Immunotactoid glomerulopathy (IGN) is a rare disease that falls into the category of Ig component deposition disorders.

## CASE PRESENTATION

A 78-year-old man with multiple medical problems, including hypertension, hyperlipidemia, dementia, and benign prostatic hypertrophy, presented with a 2-day history of anuria. The patient's son noted that he had also exhibited worsening lethargy and oliguria over the past week. The physical exam revealed a thin, slightly disoriented man in no acute distress. On admission, blood pressure was 201/96 and the patient was able to follow commands. The remainder of the physical exam was unremarkable. A Foley catheter was placed with no urine output. Pertinent initial labs included blood urea nitrogen (BUN) 136 mg/dl, creatinine (Cr) 20.1 mg/dl, estimated glomerular filtration rate (eGFR) <10 ml/min (baseline 25 mg/dl,

1.2 mg/dl, >60 ml/min, respectively, 1 week prior). The patient was hyperkalemic, with a potassium of 5.6 mmol/l, and thrombocytopenic, with a platelet count of 71,000/µl (baseline 200,000/µl, 147,000/µl 1 week prior). He was noted to have an anion gap metabolic acidosis with bicarbonate of 20.6 mmol/l and an anion gap of 18 mmol/l. There was no history of nephrotoxic agents or new medications to explain the patient's rapid decline in renal function. Initial noncontrast computed tomography (CT) revealed a right-sided lung infiltrate, possible acute cholecystitis, and a nonobstructive calculus in the parenchyma of the left kidney. Bilateral pelvic calcifications of indeterminate relationship to the distal ureters were also noted, however, no hydronephrosis was appreciated. Renal ultrasound showed bilateral medical renal disease and mild-to-moderate right midrenal arterial stenosis.

Examination of urine sediment was significant for white and red blood cells, protein, and positive for nitrite and large leukocyte esterase. During the course of hospitalization, urine culture was noted to be negative. The patient's mental status continued to worsen and urine output did not respond to multiple fluid challenges. The patient's hyperkalemia continued to worsen and hemodialysis was initiated within 24 h of presentation. Further laboratory testing was sent and findings of significance include hypocomplementemia (C3: 76 mg/dl, repeat 90 mg/dl; C4: 5 mg/dl, repeat 7 mg/dl); serum protein electrophoresis (SPEP) revealed hypoproteinemia and hypoalbuminemia, with no evidence of monoclonal spike. Urine protein electrophoresis (UPEP) was not sent secondary to anuria. Serologic tests were negative for anti-GBM antibody, Hepatitis B Surface Antigen/Antibody and Hepatitis C Antibody, antinuclear antibody (ANA), cryoglobulins, and myeloperoxidase (MPO) antibody. Hemolytic-uremic syndrome (HUS) and thrombotic thrombocytopenic purpura (TTP) workup was negative as well.

The patient underwent right kidney biopsy on his $4^{th}$ day of hospitalization, which showed multiple globally sclerosed glomeruli and severe endocapillary proliferation with neutrophil infiltrates and podocyte hyperplasia (Fig. 1). Some glomeruli showed massive eosinophilic subepithelial deposits in a global or segmental pattern (Fig. 2). Congo red stain was negative for amyloid deposits. On immunofluorescence (IF), two glomeruli showed weak staining for IgG and C3 along capillary walls (Figs. 3 and 4). Electron microscopy (EM) revealed glomeruli with large subepithethial deposits varying in size and focal subendothelial, mesangial, and podocyte cytoplasm deposits (Figs. 5 and 6). These deposits were organized and randomly arranged in nonbranching microtubules, with a thick wall and an electron-lucent core measuring 43–49 nm in diameter (Figs. 7 and 8). Deposits included nonorganized mixed granular and organized tubular types. Podocytes showed severe swelling and foot process effacement. There were no findings suggestive of acute tubular necrosis (ATN). Differential diagnosis of the biopsy included IGN, cryoglobulinemia glomerulonephritis, lupus nephritis, and acute postinfectious glomerulonephritis. The features of organized microtubules and the dominant subepithelial localization of the deposits were most consistent with a diagnosis of IGN.

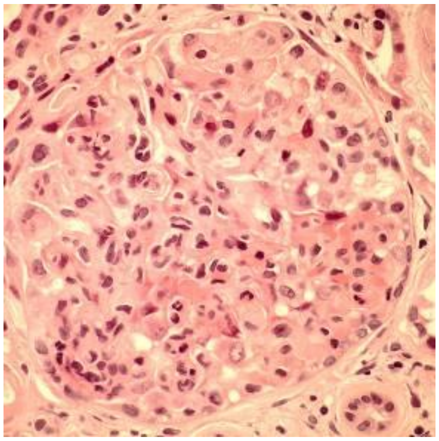

**FIGURE 1**. H&E stain: endocapillary hypercellularity with neutrophil infiltrate.

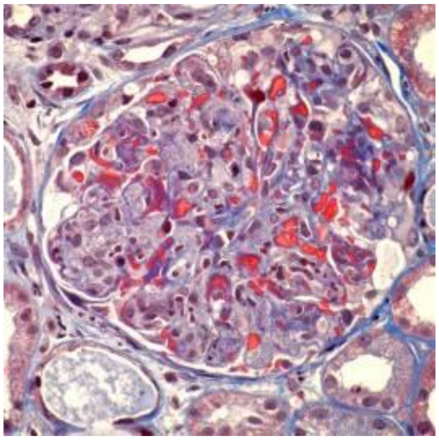

**FIGURE 2.** Trichrom stain: endocapillary hypercellularity and large subepithelial deposits (red).

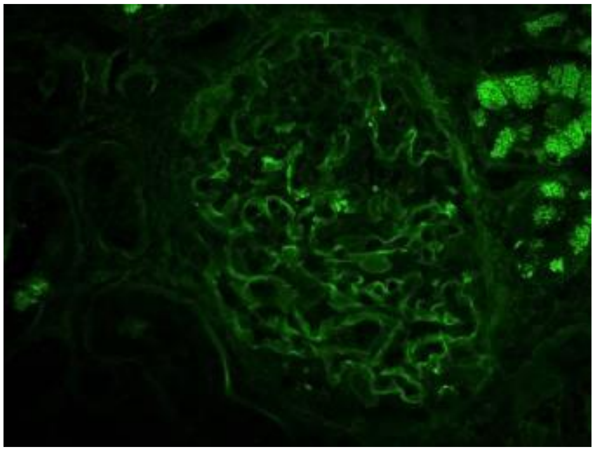

**FIGURE 3**. IF: IgG.

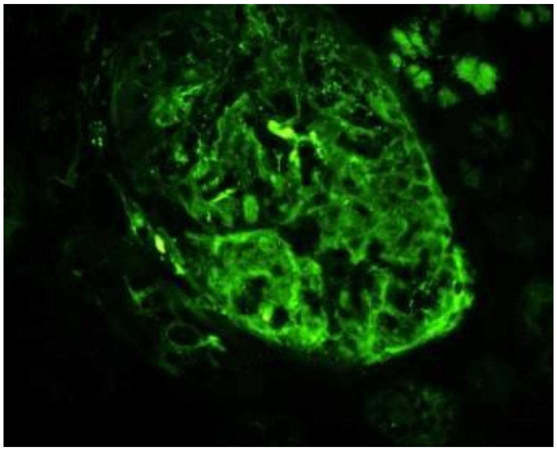

**FIGURE 4.** IF: C3.

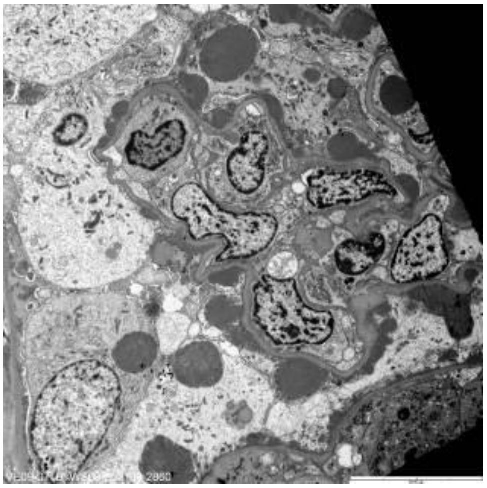

**FIGURE 5.** EM: diffuse subepithelial deposits.

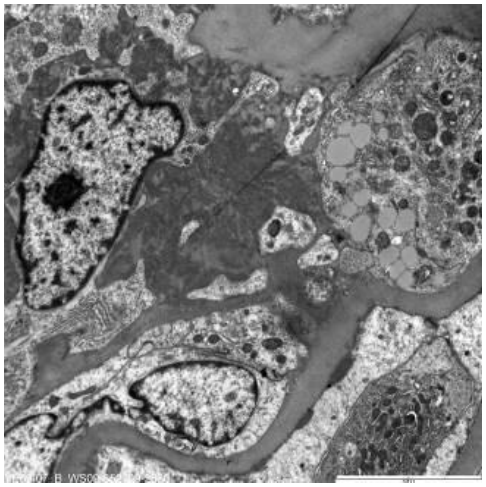

**FIGURE 6.** EM: focal mesangial deposits.

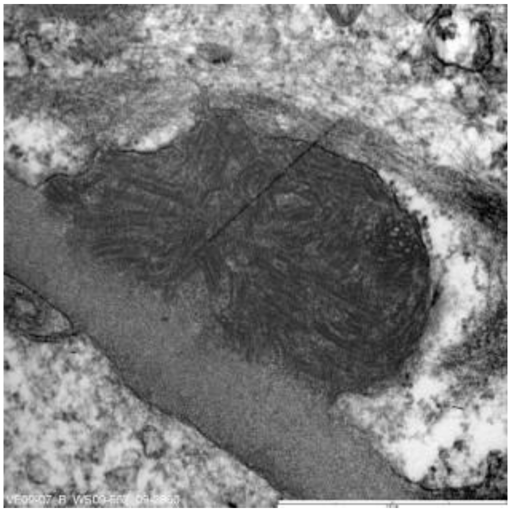

**FIGURE 7.** EM: subepithelial deposit with microtubule ultrastructure.

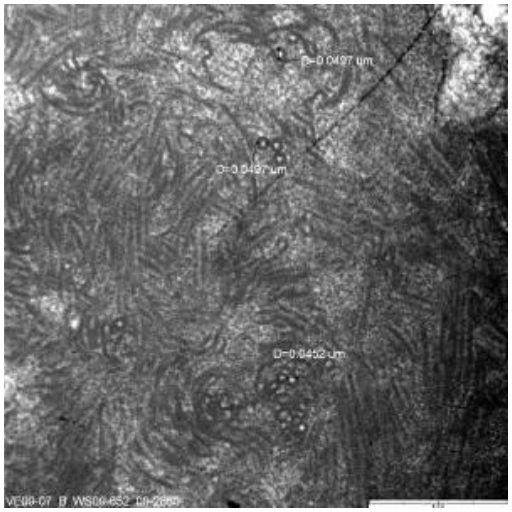

**FIGURE 8.** EM: measurements of microtubules.

## DISCUSSION

IGN is a rare disease that falls into the category of Ig component deposition disorders and EM is the gold standard for diagnosis[1,2]. It is interesting to note that unlike the other pathologies that are described in this group, the Ig components found in IGN are often not monoclonal, and in many cases, the deposits contain intact Ig molecules rather than solely light or heavy chains. IGN is categorized into this particular group due the similarities it shares with the amyloid syndromes such as the fibril-forming capacity of the pathogenic proteins. However, unlike amyloid, IGN does not stain with Congo red or thioflavine-T, and it has been postulated that many cases previously diagnosed as “Congo-red negative” amyloidosis were in fact IGN. The morphologic uniformity of the fibrils seen in individual IGN patients, as well as the presence of a lymphoproliferative disorder in some, has raised the question of whether a monoclonal component is necessary for fibril formation. It has also been suggested that the polyclonality of IGN may be due to the incorporation of Ig molecules into a fibril with a monoclonal “core”[1].

Fibrillary glomerulopathy (FGN) is considered by some to be a subset within the diagnosis of IGN due to the amyloid-like fibrils and similar EM findings. In a study of 14 IGN and nine FGN patients, clinical symptoms, including hypertension, nephrotic syndrome, and hematuria, as well as mean diameter of substructures, were similar when comparing the two pathologies. At diagnosis, there was no significant difference between the two groups when comparing age, serum Cr levels, incidence of microscopic hematuria, hypertension, and renal failure[3]. However, the two diseases have been shown to be morphologically different[3]. EM in patients with IGN show extracellular microtubule deposits arranged in densely packed parallel arrays with well-defined borders. These microtubles are >30 nm in diameter, have a central lumen, are electron lucent, and are predominantly found in the subepithelial space and mesangium. Deposition is almost always limited to the kidney. Renal biopsies of patients with FGN display randomly arranged extracellular fibrils that are ≤30 nm in diameter, with poorly defined borders and no central lumen. These deposits are unbranched and are predominantly mesangial and subepithelial as in IGN; however, in some cases, fibrils extend through the lamina densa of the GBM in patients with FGN[4].

Immunofluorescence in IGN shows circulating paraprotein or monoclonal Ig deposition in the glomeruli and is positive for IgG, C3, and κ and λ light chains. The deposits may be extensive as to give the appearance of linear IgG deposition similar to that seen in anti-GBM antibody disease. IgG4 has been shown to be the predominant IgG in deposits[3]. Immunologically, patients may have low titers of oftenspeckled ANA with normal levels of complements. As in our patient, serologic workup is usually unremarkable, although one patient in the Bridoux study did have low levels of C3 and C4 as in this patient.

## Presentation

Most cases of IGN are idiopathic. The average age of presentation is approximately 45 years, however it ranges from 10–80 years. Patients present with features similar to other primary renal diseases associated with nephrotic range proteinuria. In approximately 60–70% of cases, patients have microscopic hematuria and 50% of patients are hypertensive with renal insufficiency. In most cases, patients develop end-stage renal disease (ESRD) within 2–6 years. A study done on patients with FGN showed that the time to ESRD varied depending on histology on light microscopy. Median times were as follows[7]:

- 7 months, diffuse sclerosing
- 20 months, diffuse proliferative
- 44 months, membranoproliferative
- 80 months, mesangial proliferative/sclerosing
- 87 months, membranous

Our patient is different from all of the cases thus far presented in the literature as he progressed to ESRD within 1 week of initial symptoms. His BUN and Cr increased in this time from 25 to 136 mg/dl and 1.2 to 20 mg/dl, respectively. Of the cases of IGN cited to date, all of the patients have shown a much more prolonged progression to ESRD. Although the biopsy did not show evidence for ATN, the presence of concurrent pulmonary and biliary tract processes may have set the stage for renal ischemic injury that was not appreciated on the sampled kidney tissue.

Of note, studies have also shown that IGN can be associated with chronic lymphocytic leukemia (CLL) or B-cell lymphoma, and association with neoplasia has been shown to be more pronounced in patients with circulating monoclonal paraprotein. Hepatitis C, cryoglobulinemia, and systemic lupus erythematosus have also been shown to be associated with IGN[5]. In 1992, Alpers found that six of 11 patients with larger microtubules had associated dysproteinemia or lymphoproliferative disorder compared to one of 86 patients with smaller microtubules[6]. Rosenstock et al. showed an indistinguishable clinical picture between the two groups when separating IGN and FGN on the basis of fibril size; however, at presentation, patients with larger microtubules were more likely to have associated lymphoproliferative disorders and paraproteinemia[7]. In 1993, Fogo et al. found that patients with more organized arrays of larger microtubules were more likely to have hematopoietic disorders than patients with small, randomly oriented fibrils. In the Bridoux study, six IGN patients carried a diagnosis of CLL, one patient was diagnosed with small lymphocytic non-Hodgkin’s lymphoma, and three patients had small amounts of urinary Bence-Jones proteins[8].

## Treatment

Unfortunately, there have been no successful controlled trials to help provide guidance for appropriate therapy in patients with IGN and, thus far, no treatment has been proven to be effective. Both plasmapharesis and prednisone have been utilized with no obvious positive effects. In a study of 16 patients treated with prednisone and/or cytotoxic agents only, two patients experienced partial remission of proteinuria, with the remaining 14 patients having no response. The partial remission seen in these two patients was most likely related to a nonspecific reversal of the acute inflammatory process[9]. In the Bridoux study, 10 of 12 nephrotic IGN patients with an underlying lymphoproliferative disease and/or paraproteinemia showed complete or partial remission of nephrotic syndrome after chemotherapy was completed[3]. As most patients progress to ESRD, dialysis or renal transplantation is necessary. Recurrent disease in the graft is a common occurrence (four of eight patients in one study), but the rate of progression is typically slower than in a native kidney transplant; thus, transplantation is not contraindicated[10].

In our case, given the variable responses to treatments documented in the literature with none proven to be effective, the age of our patient, his comorbidities, and the fact that he developed ESRD, the decision was made not to treat with any cytotoxic agents/steroids.

## CONCLUSION

The differential diagnosis of IGN includes a multitude of pathologies, including cryoglobulinemia GN, lupus nephritis, acute postinfectious GN, and amyloidosis. IGN is often misdiagnosed as cryoglobulinemia as both diseases have features that overlap, such as the organized deposition of the microtubules. However, patients with IGN do not display lesions typical of cryoglobulinemia, such as dominant intracapillary hyaline thrombi or crystal inclusions within endothelial cells. A higher frequency of monoclonal Ig deposits are found in the subepithelium and intramembranously in the kidney in patients with IGN compared to cryoglobunemia as well[3]. In this case, the distinction could not be made based on morphologic features by EM. However, our patient's cryoglobulins were negative on initial and repeat testing. Lupus nephritis was excluded as ANA was negative and the patient displayed no other clinical signs of lupus. The presence of such small amounts of subendothelial deposits is also not compatible with Class IV lupus nephritis. Acute postinfectious GN specimens typically display dominant subepithelial "hump" deposits that are granular and nonorganized. The biopsy did not stain with Congo red, thus amyloidosis was ruled out. EM in this case revealed glomeruli with large subepithelial deposits of varying sizes. These deposits were randomly arranged in nonbranching microtubules with a thick wall and an electron-lucent core measuring 43–49 nm in diameter. The deposits also included nonorganized mixed granular and tubular types. These features were pathognomonic for IGN.

IGN is a diagnosis that is often missed or overlooked as it presents in a similar manner to many other renal diseases and cannot be confirmed without biopsy. Our patient presented with IGN that progressed to ESRD rapidly. Thus far in the literature, IGN and FGN have been described by a subacute progression to ESRD ranging from 7 months to 10 years. This is the first case reported of a patient with a diagnosis of IGN who developed acute kidney injury (AKI) and ESRD within 1 week of initial presentation.
